# Few‐shot learning for highly accelerated 3D time‐of‐flight MRA reconstruction

**DOI:** 10.1002/mrm.70072

**Published:** 2025-09-10

**Authors:** Hao Li, Mark Chiew, Iulius Dragonu, Peter Jezzard, Thomas W. Okell

**Affiliations:** ^1^ Centre for Integrative Neuroimaging, FMRIB Division, Nuffield Department of Clinical Neurosciences University of Oxford Oxford UK; ^2^ Physical Sciences, Sunnybrook Research Institute Toronto Ontario Canada; ^3^ Department of Medical Biophysics University of Toronto Toronto Ontario Canada; ^4^ Research & Collaborations GB&I Siemens Healthcare Ltd Camberley UK

**Keywords:** data synthesis, deep learning, few‐shot learning, image reconstruction, magnetic resonance angiography, time‐of‐flight

## Abstract

**Purpose:**

To develop a deep learning‐based reconstruction method for highly accelerated 3D time‐of‐flight MRA (TOF‐MRA) that achieves high‐quality reconstruction with robust generalization using extremely limited acquired raw data, addressing the challenge of time‐consuming acquisition of high‐resolution, whole‐head angiograms.

**Methods:**

A novel few‐shot learning‐based reconstruction framework is proposed, featuring a 3D variational network specifically designed for 3D TOF‐MRA that is pre‐trained on simulated complex‐valued, multi‐coil raw k‐space datasets synthesized from diverse open‐source magnitude images and fine‐tuned using only two single‐slab experimentally acquired datasets. The proposed approach was evaluated against existing methods on acquired retrospectively undersampled in vivo k‐space data from five healthy volunteers and on prospectively undersampled data from two additional subjects.

**Results:**

The proposed method achieved superior reconstruction performance on experimentally acquired in vivo data over comparison methods, preserving most fine vessels with minimal artifacts with up to eight‐fold acceleration. Compared to other simulation techniques, the proposed method generated more realistic raw k‐space data for 3D TOF‐MRA. Consistently high‐quality reconstructions were also observed on prospectively undersampled data.

**Conclusions:**

By leveraging few‐shot learning, the proposed method enabled highly accelerated 3D TOF‐MRA relying on minimal experimentally acquired data, achieving promising results on both retrospective and prospective in vivo data while outperforming existing methods. Given the challenges of acquiring and sharing large raw k‐space datasets, this holds significant promise for advancing research and clinical applications in high‐resolution, whole‐head 3D TOF‐MRA imaging.

## 1 INTRODUCTION

Angiography, the imaging of blood vessels, is particularly vital in the brain, where ischemia or hemorrhage can have severe clinical consequences.^1^ Cerebral angiograms provide critical diagnosis and treatment planning information for various cerebrovascular diseases, including stroke,^2^ steno‐occlusive disease,^3^ and arteriovenous malformation.^4^ However, conventional angiographic techniques, such as digital subtraction angiography, rely on X‐ray imaging and require contrast agent injection, exposing patients to ionizing radiation and limiting their use in pediatric or longitudinal examinations.^5^ Although contrast‐enhanced MRA (CE‐MRA) offers a radiation‐free alternative, concerns have been raised regarding the accumulation of gadolinium‐based contrast agents.^6^

Time‐of‐flight (TOF) MRA is a non‐contrast technique that exploits the magnetization differences between stationary tissue and inflowing blood.^7^ With multiple overlapping thin‐slab acquisitions,^8^ 3D TOF‐MRA provides excellent visualization of the arterial vasculature and is routinely used in clinical assessments of cerebrovascular diseases.^7^ High‐resolution, whole‐head 3D TOF‐MRA could enable more comprehensive cerebrovascular assessments, improving diagnostic accuracy for small aneurysms^9^, ^10^ and cerebral small vessel diseases.^11^ However, acquiring high‐resolution whole‐brain 3D TOF‐MRA requires long scan times, which pose challenges in clinical workflows. Prolonged acquisitions also increase patient discomfort and susceptibility to motion‐related artifacts, potentially compromising image quality and diagnostic reliability.^12^

Parallel imaging (PI) techniques, such as GRAPPA^13^ and SENSE,^14^ are commonly employed to reduce scan times by leveraging the spatial sensitivity of multiple receiver coils to reconstruct undersampled k‐space data. However, PI reconstructions typically degrade at acceleration factors beyond 3–4 due to noise amplification and residual artifacts. Compressed sensing (CS) has been explored extensively for 3D TOF‐MRA,^15^, ^16^, ^17^ utilizing incoherent undersampling and sparsity constraints to achieve higher acceleration factors.^18^ However, CS reconstructions require lengthy processing times with careful parameter tuning, limiting their clinical application.^19^ More recently, wave‐controlled aliasing in parallel imaging (wave‐CAIPI)^20^ has been applied to 3D TOF‐MRA,^21^ demonstrating improved image quality at higher acceleration factors compared to conventional PI and CS. However, wave‐CAIPI necessitates complex implementation and careful optimization of wave gradient parameters to minimize flow‐related artifacts.^21^

Deep learning (DL)‐based reconstruction methods have emerged as powerful alternatives for accelerating MRI, consistently outperforming CS while significantly reducing reconstruction times.^22^, ^23^, ^24^ Among these, model‐based DL approaches that integrate the MRI forward model into unrolled iterative optimization frameworks have demonstrated particularly strong performance.^25^, ^26^ For example, the end‐to‐end variational network (E2E‐VarNet)^26^ employs 2D U‐Nets^27^ to learn gradients within unrolled optimization steps. However, most DL‐based methods are applied to 2D k‐space data due to memory and data availability limitations, which may not be optimal for 3D TOF‐MRA, where vessel signals are inherently connected across adjacent slices. Additionally, training such models typically requires large raw k‐space datasets, which are generally unavailable for 3D TOF‐MRA. Consequently, most previous studies have relied on small in‐house datasets.^28^, ^29^, ^30^, ^31^

For reconstructing undersampled multi‐coil 3D TOF‐MRA data, Jun et al. proposed a 3D multi‐stream convolutional neural network (CNN) called DPI‐Net, which reconstructs directly in the image domain.^28^ Chung et al. developed a multiplanar 2D OT‐cycleGAN that enables training with unmatched reference data, achieving reconstruction results on an in‐house dataset that were comparable to supervised learning approaches trained on paired raw data.^29^ Most recently, Sun et al. introduced an uncertainty‐aware reconstruction model for accelerated 7T TOF‐MRA, utilizing evidential deep learning to support uncertainty quantification.^32^

Data limitations pose significant challenges for effectively training and validating these DL models, which may fail to generalize to different populations, anatomical variations, protocol settings, and scanners. Small datasets also increase the risk of overfitting, reducing robustness and reproducibility across institutions.^24^ While self‐supervised learning has been proposed to address data limitations by leveraging undersampled data, it still commonly requires large undersampled k‐space datasets.^33^, ^34^, ^35^ Emerging zero‐shot self‐supervised methods^36^ attempt to bypass this requirement, but they involve lengthy per‐reconstruction training times, making them impractical for large‐scale clinical deployment.

To overcome these challenges and minimize the burden of acquiring large local datasets and conducting extensive retraining, this work proposes a data‐ and time‐efficient DL‐based reconstruction framework for highly accelerated 3D TOF‐MRA that integrates few‐shot learning. The framework eliminates the need for large amounts of experimental raw k‐space data by leveraging large‐scale pre‐training on diverse simulated data, followed by fine‐tuning on minimal site‐specific raw data. Specifically, a 3D reconstruction model adapted from E2E‐VarNet is first pre‐trained using simulated raw k‐space data generated from diverse publicly available magnitude images. Fine‐tuning is then performed using only two experimentally acquired fully sampled in vivo single‐slab datasets. The proposed method was evaluated against established methods on retrospectively undersampled in vivo multi‐slab k‐space acquisitions from healthy subjects scanned at 3T. Additionally, the effectiveness of the proposed simulation pipeline was assessed in comparison to other raw k‐space data synthesizing techniques. Finally, additional prospectively undersampled acquisitions were used to assess the proposed method's robustness in a real‐world clinical scenario. This work builds upon an accepted abstract for presentation at the 2025 ISMRM Annual Meeting.^37^

## 2 METHODS

**FIGURE 1:**
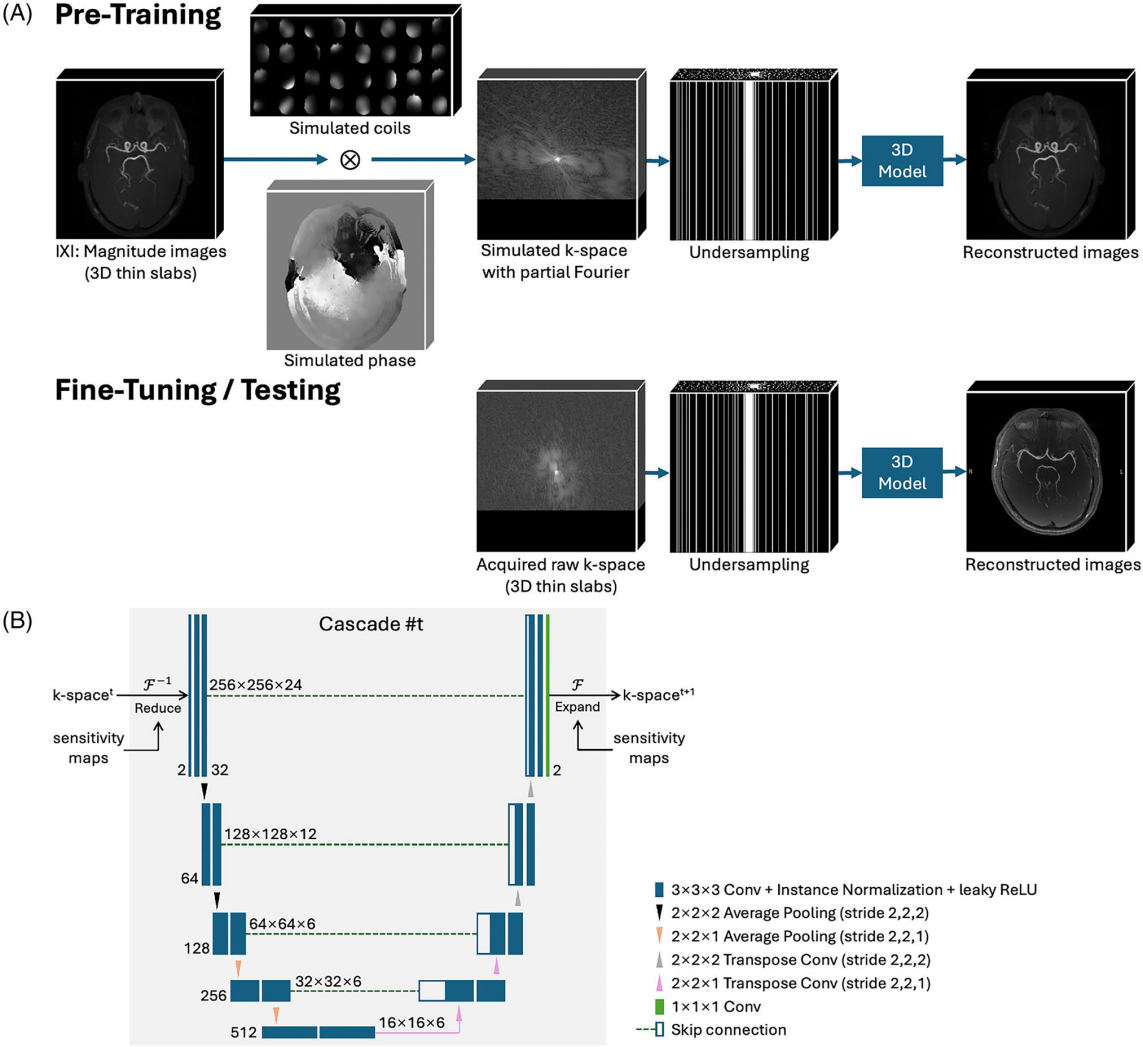
(A) Proposed few‐shot learning framework. (B) Proposed model architecture in each cascade in E2E‐VarNet. To overcome data limitations, large‐scale open‐source magnitude images from the IXI dataset were used to simulate complex‐valued multi‐coil k‐space data. Partial Fourier and Poisson‐disc undersampling were then applied. The proposed model integrates 3D pooling and convolution with customized down/up‐sampling for thin‐slab inputs. After pre‐training on simulated k‐space data, the model underwent fine‐tuning using two single‐slab in vivo datasets acquired from a 3T scanner. The final model was evaluated on additional experimentally acquired multi‐slab in vivo k‐space datasets.

To effectively train a DL model capable of reconstructing complex‐valued multi‐coil raw k‐space, phase and coil sensitivity maps were simulated to leverage magnitude 3D TOF‐MRA images from the large open‐source multi‐center multi‐vendor IXI dataset (https://brain‐development.org/ixi‐dataset/). An overview of this approach is depicted in Figure 1.

### 2.1 Raw k‐space data simulation

Previous studies have explored various approaches for phase synthesis, including physics‐based modeling,^38^ sinusoidal functions,^39^ and conditional generative adversarial networks (GANs).^40^ However, GAN‐based methods require large raw k‐space training datasets, which are generally unavailable for 3D TOF‐MRA. Recent efforts have instead leveraged widely available natural images and videos to train DL models for MRI reconstruction, demonstrating promising results. Notably, Wang et al.^41^ simulated the phase by applying Fourier truncation to random complex white Gaussian noise following the LORAKS method.^42^ However, their approach borrowed coil sensitivity maps from the 2D fastMRI dataset^22^, ^23^, ^43^ that typically includes at most 16 coil channels with different coverages, which is challenging to adapt to 3D TOF‐MRA. Meanwhile, Jaubert et al.^44^ simulated coil sensitivity maps using 2D Gaussians with randomized maximum intensity, SDs, and centers. Also, they simulated phase variations relating to specific structures in natural images using their RGB channels, which is not applicable for simulating structure‐related phase variations of grayscale MRI images.

In this work, to simulate the phase ϕ realistically, the conjugate symmetric k‐space of the magnitude image is corrupted by multiple modulated noise components, resulting in a non‐symmetric k‐space from which the phase is extracted. The magnitude provides structures to the phase, as observed in experimental data, while the spatially modulated noise components simulate low‐frequency background phase variations due to inhomogeneities, and various sources of noise.

The phase simulation model is proposed as:

(1) $$\begin{array}{cc} & \phi \left(r\right)=\arg\left(\left|x\left(r\right)\right|+{ℱ}^{-1}\left\{\sum_{j=1}^{N_{\mathrm{phs}}}W\left(k;\sigma_{G_{j}}\right)\cdot n_{j}\left(k\right)\right\}\right),\\ & \;n_{j}∼𝒩\left(0,\sigma_{n_{j}}^{2}\right) \end{array}$$

Where:

- ${ℱ}^{-1}$: The inverse Fourier transform.
- |x(r)|: The magnitude image with the 3D spatial coordinate $r=\left(r_{x},r_{y},r_{z}\right)$.
- $n_{j}\left(k\right)$: A random complex‐valued white Gaussian noise at each $k=\left(k_{x},k_{y},k_{z}\right)$ for the j‐th noise component, sampled from a Gaussian distribution with zero mean and variance $\sigma_{n_{j}}^{2}$.
- $W\left(k;\sigma_{G_{j}}\right)$: The Gaussian weight map for the j‐th noise component, centered at (0, 0,0). W is the Gaussian function, where the SD $\sigma_{G_{j}}$ controls the spread of the Gaussian, thereby modulating the spatial frequency of the variations introduced by the noise component.
- $N_{\mathrm{phs}}$: The total number of distinct noise components added to the phase simulation.

The parameters $\sigma_{n_{j}}$ and $\sigma_{G_{j}}$ were randomized for each dataset to capture the necessary diversity of variations and were empirically optimized based on reconstruction performance on the validation set, with their specific values and ranges provided in Table S1 of the Supporting Information document. To avoid a phase bias toward zero, the amplitudes of the noise components are set to exceed those of the original signal from the magnitude image.

**FIGURE 2:**
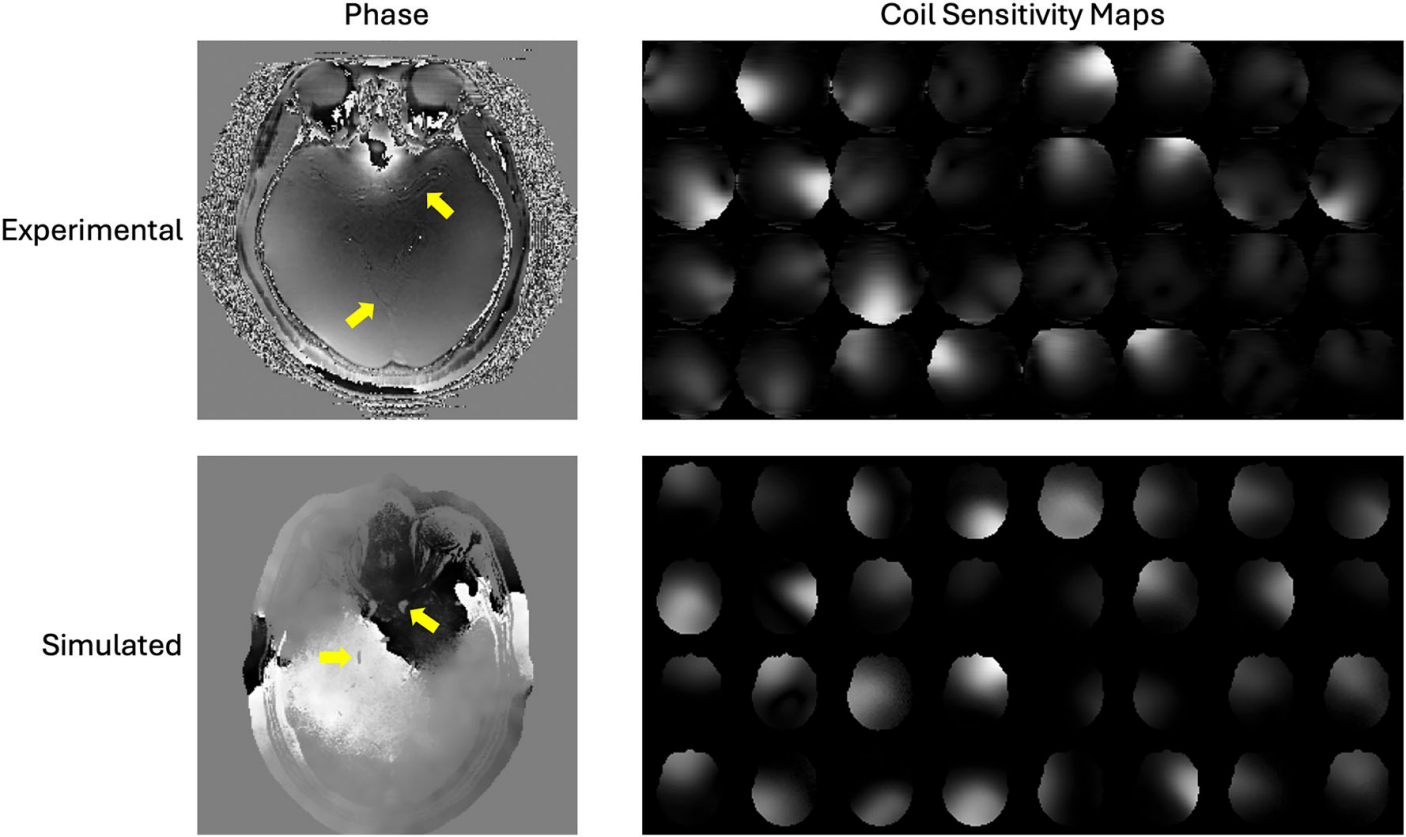
Comparison of simulated phase and coil sensitivity maps with experimentally acquired phase and coil sensitivity maps from in vivo data. Yellow arrows indicate structures in the experimental and simulated phase images that coincide with vessels.

A representative simulated phase image is shown in Figure 2 alongside the genuine phase from experimental in vivo data. The simulated phase demonstrates similar characteristics to the genuine phase, exhibiting slowly varying phase across the head, along with more detailed variations that coincide with vessels and other anatomical structures.

To simulate coil sensitivity maps, a head mask $M_{\text{head}}\left(r\right)$ was first extracted using Otsu's thresholding followed by morphological operations, including binary erosion with four iterations and binary dilation with nine iterations. The boundary coordinates are then identified using the “find_boundaries” function from the scikit‐image library (https://scikit‐image.org/). A center location $c=\left(c_{x},c_{y},c_{z}\right)$ is randomly sampled from the boundaries using a uniform distribution 𝒰(boundaries) for each coil. For the i‐th coil, a multivariate spatial Gaussian $G_{i}$ centered at $c_{i}$ is generated and randomly rotated about the *z*‐axis, where $\sigma_{x_{i}}$, $\sigma_{y_{i}}$, and $\sigma_{z_{i}}$ control its spread and shape and are randomly sampled for each coil.

The final sensitivity map for the i‐th coil is computed as:

(2) $$\begin{array}{cc} & S_{i}\left(r\right)=\mathrm{dSS}\left(G_{i}\left(r\right)+{ℱ}^{-1}\left\{\sum_{k=1}^{N_{\mathrm{csm}}}W\left(k;\sigma_{G_{k}}\right)\cdot n_{k}\left(k\right)\right\}\right),\\ & \;\;n_{k}∼𝒩\left(0,\sigma_{n_{k}}^{2}\right), \end{array}$$

where $N_{\mathrm{csm}}$ modulated noise components are added to simulate low‐frequency background variations and sources of measurement noise, and dSS normalizes the map by the sum‐of‐squares across coils. Although this formulation is similar to Eq. (1), instead of extracting the phase using arg, it simulates the complex‐valued low‐spatial‐frequency coil sensitivity maps directly and adds independent noise to each coil, a critical step as applied in previous work.^44^ The Gaussian parameters are listed in Table S2 in the Supporting Information document and representative simulated coil sensitivity maps are shown in Figure 2.

After combining the magnitude image with the simulated phase and 32 coil sensitivity maps, partial Fourier (PF) in the readout direction was simulated, as used in most 3D‐TOF‐MRA protocols to reduce the TE. To further diversify the dataset, random bias fields caused by field inhomogeneities were introduced using the “RandomBiasField” function (with coefficients = 0.3 and order = 3) from the TorchIO library.^45^ Subsequently, 2D pseudorandom variable‐density Poisson‐disc undersampling masks with a fully sampled calibration region (12 × 6) in the k‐space center were applied in the *k*_*y*_‐*k*_*z*_ planes.^46^ All simulation processes, excluding the Poisson‐disc undersampling, were computed once per dataset and then fixed throughout the training period. For each training run, a fixed acceleration rate was used, while the Poisson‐disc undersampling mask was randomized each time a dataset was loaded, enabling the model to generalize across different undersampling patterns.

### 2.2 Model modifications

To reconstruct 3D TOF‐MRA data, where vessel signals exhibit strong continuity across slices, this work extends the physics‐informed 2D E2E‐VarNet^26^ by implementing 3D pooling and convolution operations throughout all U‐Nets. This modification enables the model to effectively capture both intra‐ and inter‐slice correlations, enhancing its ability to reconstruct the complex 3D vasculature in 3D TOF‐MRA. Compared to the previous work that used DPI‐Net,^28^ which utilizes a 3D CNN to learn image‐to‐image mapping, the proposed model is expected to have improved performance and generalizability by incorporating an MRI forward model.

To further optimize the model for the multiple thin‐slab data used in 3D TOF‐MRA, its downsampling (max‐pooling) and upsampling (up‐convolution) layers were carefully designed. Specifically, four downsampling levels were used along the longer in‐plane axes (*x* and *y*), while only two levels were applied along the shorter slice axis to mitigate excessive loss in the through‐plane dimension. This architectural modification ensures that sufficient structural information is retained at the bottleneck of the U‐Net across the much smaller slice dimension, preventing the degradation of fine vascular details. Additionally, PF constraints were enforced during each data consistency step by ensuring the asymmetric PF region in k‐space remained identical to the acquired signals, aligning with the readout PF acquisition commonly employed in 3D TOF‐MRA protocols.

### 2.3 Training data and in vivo acquisition

The simulated training data were derived from the IXI dataset (https://brain‐development.org/ixi‐dataset/), comprising images from 341 subjects (153 males, 188 females; mean age 49.20 ± 16.78 y) acquired with a 3T Philips Intera MRI scanner (Philips Medical Systems, Best, The Netherlands), a 1.5T Philips Gyroscan Intera MRI scanner (Philips Medical Systems, Best, The Netherlands), and a 1.5T GE MRI scanner (GE HealthCare, Wisconsin, United States) at Hammersmith Hospital, Guy's Hospital, and the Institute of Psychiatry in London, United Kingdom. The imaging parameters, as obtained from the dataset's official website and NIfTI headers, were as follows: TR = 16.7 ms (3T) or 20 ms (1.5 T); TE = 5.8 ms (3 T) or 6.9 ms (1.5 T); flip angle = 16° (3T) or 25° (1.5T); acquisition matrix = 288 × 286; voxel resolution = 0.8 × 0.8 × 0.8 mm^3^; and number of slices = 100. Each 3D magnitude image dataset was divided into five overlapping slabs along the slice axis to replicate multi‐slab acquisition, resulting in 24 slices per slab. The simulated data derived from the IXI dataset was used exclusively as the training set for pre‐training.

To evaluate the proposed DL‐based reconstruction method on retrospectively undersampled raw k‐space data, five healthy subjects (Cohort 1; four males, one female; mean age 32.06 ± 7.04 y) were scanned on a 3T Siemens Prisma scanner (Siemens Healthineers, Erlangen, Germany) using a 32‐channel receive head coil under a technical development protocol approved by local ethics and institutional committees. The multi‐slab 3D TOF‐MRA sequence parameters were similar to the IXI data, as follows: TR = 18 ms; TE = 3.8 ms; flip angle = 16°; acquisition matrix = 288 × 288; voxel resolution = 0.8 × 0.8 × 0.8 mm^3^; and PF = 26% in the readout direction. For each subject, five fully sampled slabs, each consisting of 24 slices with a 20% overlap, were acquired in a total scan time of 12 min and 22 s.

Additionally, two single‐slab datasets were acquired from two other healthy volunteers (Cohort 2; two males; mean age 27.90 ± 2.09 y) on the same scanner as a validation set for model training. One of these datasets was later used for fine‐tuning, while the remaining dataset served as an independent validation set. The single‐slab sequence parameters were as follows: TR = 21 ms; TE = 3.48 ms; flip angle = 18°; acquisition matrix = 256 × 256; voxel resolution = 0.8 × 0.8 × 1.0 mm^3^; PF factor = 29%; number of slices = 20; and acquisition time = 1 min and 52 s.

Finally, prospectively undersampled datasets were acquired using a modified 3D TOF‐MRA sequence with predefined pseudorandom variable‐density Poisson‐disc undersampling masks, using the same imaging parameters as Cohort 1, to demonstrate the real‐world potential of this approach. An eight‐times accelerated acquisition (scan time 1 min 47 s), along with a fully sampled reference (scan time 12 min 22 s), was obtained from five healthy volunteers (Cohort 3; four males, one female; mean age 24.72 ± 2.20 y). As the acceleration factor is defined by the undersampling in k‐space or, equivalently, the reduction factor in the number of readouts, the slight deviation from an exact 8× reduction in actual scan time results from additional scan overhead, including pre‐scans for system calibration.

### 2.4 Experiments

To evaluate the reconstruction performance of the proposed method on experimentally acquired in vivo 3D TOF‐MRA data, a combination of quantitative metrics was used, including the peak SNR (PSNR),^47^ structural similarity index measure (SSIM),^48^ normalized mean square error (NMSE), and the vessel‐masked SSIM (VM‐SSIM). PSNR, SSIM, and NMSE were computed by comparing the entire 3D reconstructed volume with the corresponding fully sampled reference and averaging the results across the dataset. VM‐SSIM was computed from axial maximum intensity projection (MIP) images using vessel masks derived from the fully sampled reference as described by previous studies,^21^, ^46^ which focuses evaluation on vascular structures and has been shown to correlate strongly with visual assessments by radiologists.^46^, ^49^ By including both global and vessel‐specific measures, our evaluation aims to capture not only the overall image fidelity but also the accuracy of vascular morphology, which is critical for the clinical utility of TOF‐MRA. All fully sampled references in this study were obtained by combining the coil images using the conjugate of coil sensitivity maps estimated by ESPIRiT,^50^ implemented based on the SigPy package (https://github.com/mikgroup/sigpy).

The proposed method was compared to zero‐filling, L1 wavelet‐regularized CS,^18^ and several DL‐based methods, namely: (i) Few‐shot DPI‐Net (pre‐trained and fine‐tuned using the proposed few‐shot learning framework for 3D TOF‐MRA)^28^; (ii) Original 2D E2E‐VarNet (originally pre‐trained on experimental 2D T1w, T2w, FLAIR raw k‐space data from the fastMRI dataset)^26^; (iii) Fine‐tuned original 2D E2E‐VarNet (the original pre‐trained model fine‐tuned using two single‐slab experimental 3D TOF‐MRA datasets); and (iv) Few‐shot 2D E2E‐VarNet (pre‐trained and fine‐tuned using the proposed few‐shot learning framework for 3D TOF‐MRA). The proposed 3D model contains 84.6 million parameters, whereas DPI‐Net has 392 thousand parameters, and each 2D E2E‐VarNet variant has 29.9 million parameters. For 2D networks, 2D k‐space slices were obtained by inverse Fourier transformation along the readout direction. Before image reconstruction, each 32‐channel k‐space dataset was first cropped to a standard in‐plane size of 256 × 256 and compressed into eight virtual coils to reduce the computational burden using an SVD‐based method.^51^

All models were pre‐trained for 50 epochs for each undersampling factor (4× and 8×) using Adam optimizers, a 0.0003 learning rate, validation‐based early stopping, and L1 + SSIM loss minimization on an NVIDIA A100 80GB GPU. The proposed 3D model took approximately 3.5 days to pre‐train, while 2D models took around 1.5 days. Fine‐tuning on an individual experimental single‐slab dataset from the validation set (Cohort 2) followed the same protocol as pre‐training (50 epochs, learning rate = 0.0003, validation‐based early stopping, and L1 + SSIM loss). Fine‐tuning required approximately 10 min for 3D models and 8 min for 2D models. The early stopping criterion within the 50‐epoch limit was sufficient to ensure all models converged effectively without overfitting, enabling a fair comparison despite differences in model size and training time. This convergence behavior during fine‐tuning of the proposed method is illustrated in Figure S1 of the Supporting Information.

To further assess the proposed method, ablation studies were conducted. First, the proposed 3D model was trained using only two single‐slab experimental 3D TOF‐MRA datasets—one for fine‐tuning and one for validation—without any pre‐training on simulated raw k‐space data (named Proposed w/o pre‐training) to evaluate the importance of pre‐training. Second, the proposed model was pre‐trained on simulated data but was not fine‐tuned (named Proposed w/o fine‐tuning) to assess the impact of fine‐tuning. Third, the model was fine‐tuned using experimentally acquired single‐slab data, but only the magnitude component was retained, while simulated phase and coil sensitivity maps were used in place of the experimental ones (denoted Proposed w/o exp. fine‐tuning). This was done to investigate whether performance gains arose primarily from the use of more realistic experimental raw data or from reducing the domain gap between the simulated multi‐vendor training data and the site‐specific acquisition. Lastly, the proposed model architecture was tested without the customized downsampling and upsampling (denoted Proposed w/o modified pooling) to assess the impact of architectural modifications aimed at preserving more through‐plane features for thin‐slab 3D TOF‐MRA data.

To evaluate the impact of the proposed raw k‐space data simulation approach, the proposed 3D model was pre‐trained on two alternative datasets generated using recent methods. Specifically, these two datasets replaced part of the proposed simulation pipeline with the phase simulation method from LORAKS,^42^ as recently used by Wang et al.,^41^ and the recent coil sensitivity map simulation method from Jaubert et al.,^44^ respectively.

Finally, to assess the performance and robustness of the proposed method on prospectively undersampled data, reconstructed images were aligned to their fully sampled references using rigid registration with FLIRT (FMRIB's Linear Image Registration Tool)^52^, ^53^ from the FSL library (https://fsl.fmrib.ox.ac.uk/) to correct for inter‐scan misalignment.

## 3 RESULTS

### 3.1 Comparison of retrospective dataset reconstructions

**FIGURE 3:**
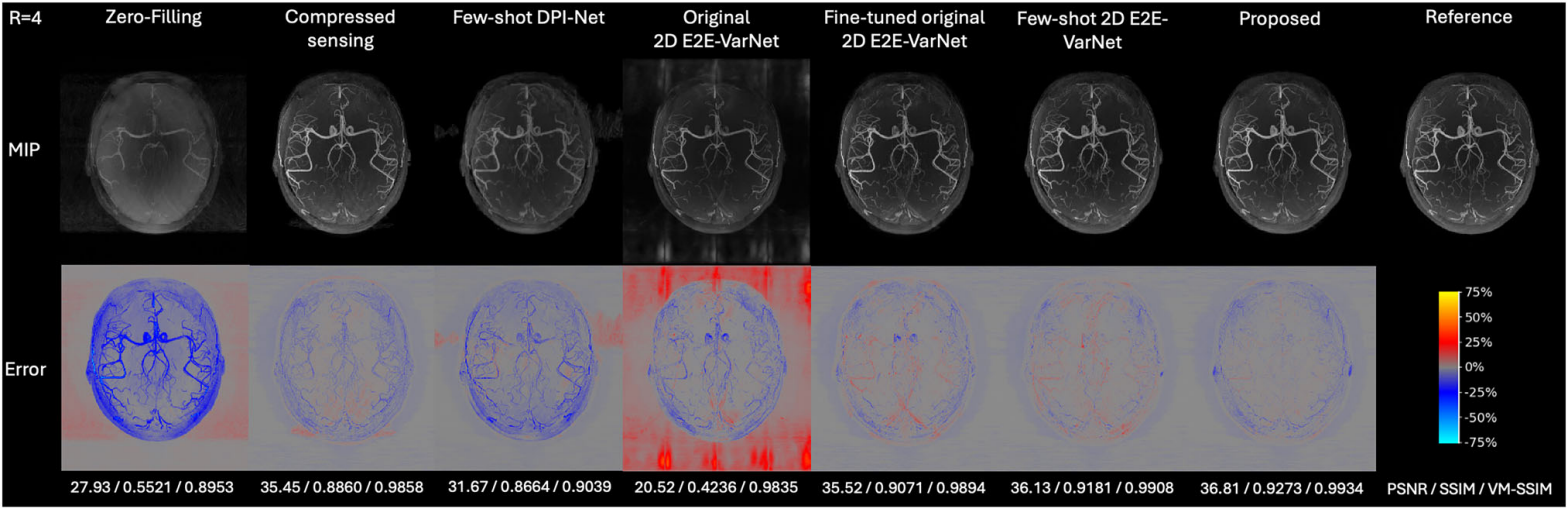
Axial MIP reconstructions of retrospectively undersampled experimental in vivo data for acceleration factor *R* = 4, showing results for comparison methods alongside the fully sampled reference. The top row shows reconstructed MIP angiograms (greyscale‐adjusted for comparison), the second row shows error maps (percentage difference from the reference normalized by the maximum intensity), and PSNR/SSIM/VM‐SSIM metrics are reported at the bottom.

**FIGURE 4:**
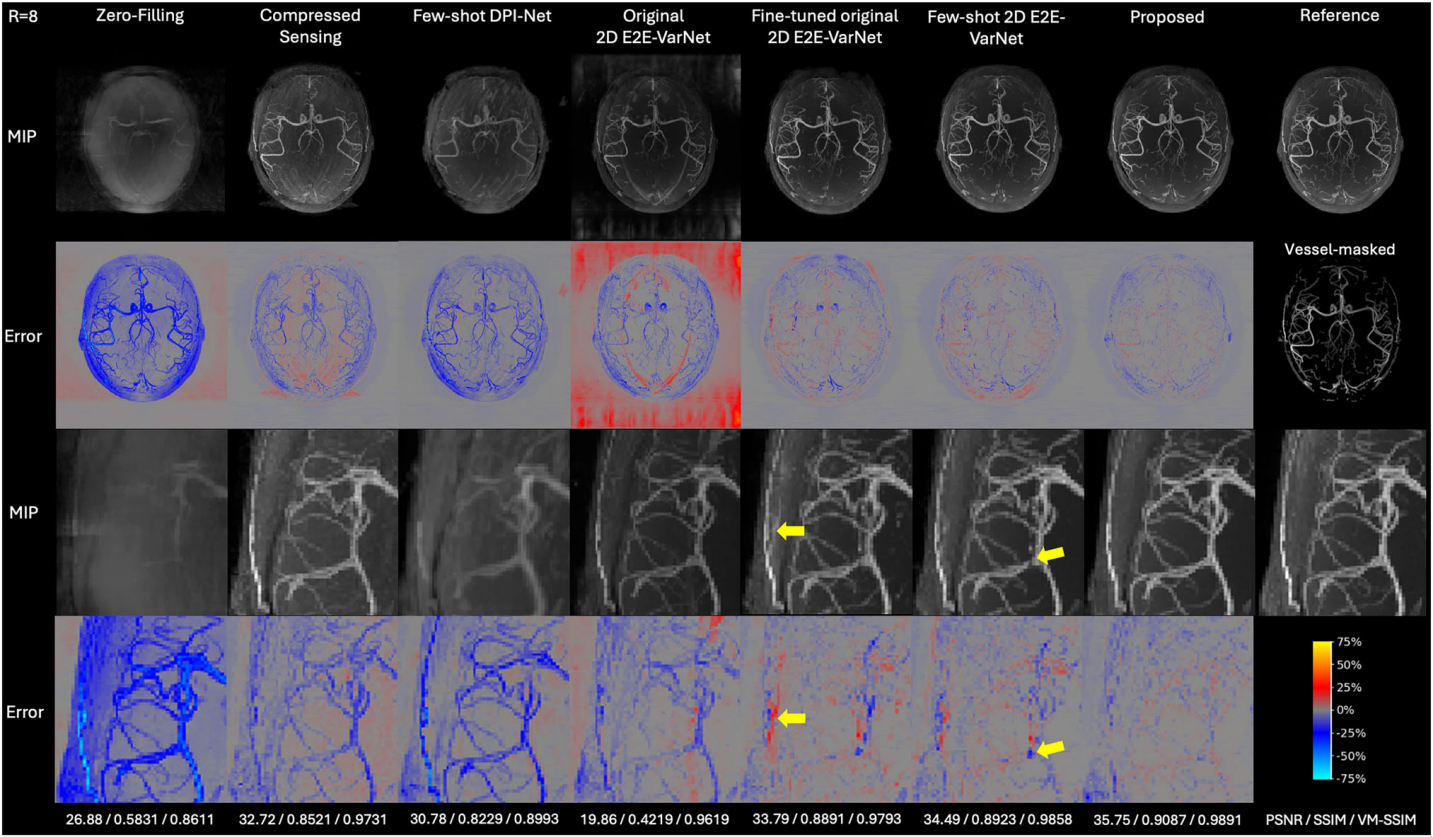
Axial MIP reconstructions of retrospectively undersampled experimental in vivo data for *R* = 8 with zoomed views. The first two rows follow Figure 3, the third and fourth rows are zoomed views of the first two rows, and PSNR/SSIM/VM‐SSIM metrics are shown at the bottom. The vessel‐masked MIP, generated using the vessel mask for VM‐SSIM computation, is shown below the reference. Yellow arrows indicate artifacts in Fine‐tuned original 2D E2E‐VarNet and missed vessel signals in Few‐shot 2D E2E‐VarNet as examples.

Two acceleration factors, *R* = 4 and *R* = 8, were tested retrospectively on the experimentally acquired in vivo k‐space data, with representative axial MIP reconstructions shown in Figure 3 and Figure 4, and additional representative reconstructions from another subject provided in Figures S2 and S3 in the Supporting Information. Reconstruction times for each multi‐slab dataset on an NVIDIA H100 10GB GPU were approximately 125 s for CS, 12 s for Few‐shot DPI‐Net, and 15 s for both 2D E2E‐VarNets and the proposed method.

The proposed method demonstrated superior image quality, retaining most fine vessels with minimal noise and artifacts, especially for *R* = 8. In contrast, other methods exhibited higher noise and aliasing, particularly Original 2D E2E‐VarNet and Fine‐tuned original 2D E2E‐VarNet. However, pre‐training on the simulated 3D TOF‐MRA raw k‐space dataset via the proposed few‐shot learning framework significantly enhanced the performance of 2D E2E‐VarNet, though some aliasing artifacts still remained. Few‐shot DPI‐Net exhibited a relatively large degradation in reconstruction quality from *R* = 4 to *R* = 8, with noticeable blurring and missing vessels.

Quantitative results in Table 1 further underscore the superior performance of the proposed method, especially for *R* = 8, demonstrating a 30.1% reduction in NMSE, along with improvements of 1.77 dB in PSNR, 2.66% in SSIM, and 1.03% in VM‐SSIM compared to the Fine‐tuned original 2D E2E‐VarNet. Interestingly, although the Original 2D E2E‐VarNet produced the lowest PSNR and SSIM—even lower than zero‐filling—due to substantial aliasing and artifacts mostly in the background, it attained a relatively high VM‐SSIM, just below that of the CS baseline. This observation aligns with the MIP reconstructions in Figure 3 and Figure 4, where vessels reconstructed by the Original 2D E2E‐VarNet are distinct despite surrounding artifacts, in contrast to the blurred or missing vessels seen in zero‐filling and Few‐shot DPI‐Net.

**TABLE 1:** Quantitative comparison of reconstruction performance on retrospectively undersampled experimental in vivo data for *R* = 4 and *R* = 8, showing results for methods in Figure 3 and Figure 4.

Acceleration	Metric	Zero‐filling	CS	Few‐shot DPI‐Net	Original 2D E2E‐VarNet	Fine‐tuned original 2D E2E‐VarNet	Few‐shot 2D E2E‐VarNet	Proposed
*R* = 4	PSNR ↑	27.39 ± 1.13	35.11 ± 1.36	30.46 ± 1.71	20.54 ± 0.87	35.03 ± 1.40	35.64 ± 1.44	**36.39 ± 1.36**
	SSIM ↑	0.5454 ± 0.0382	0.8869 ± 0.0128	0.8194 ± 0.0530	0.4366 ± 0.0158	0.9015 ± 0.0142	0.9135 ± 0.0128	**0.9201 ± 0.0104**
	VM‐SSIM ↑	0.8882 ± 0.0208	0.9823 ± 0.0031	0.8967 ± 0.0234	0.9736 ± 0.0103	0.9854 ± 0.0056	0.9885 ± 0.0026	**0.9921 ± 0.0036**
	NMSE ↓	0.1762 ± 0.0271	0.0436 ± 0.0044	0.1313 ± 0.0322	0.3197 ± 0.0755	0.0413 ± 0.0077	0.0386 ± 0.0069	**0.0314 ± 0.0044**
*R* = 8	PSNR ↑	26.79 ± 1.04	31.27 ± 1.54	28.99 ± 1.75	19.61 ± 0.91	33.35 ± 1.57	33.55 ± 1.64	**35.12 ± 1.47**
	SSIM ↑	0.5673 ± 0.0407	0.8530 ± 0.0291	0.8073 ± 0.0415	0.4285 ± 0.0130	0.8801 ± 0.0210	0.8851 ± 0.0193	**0.9067 ± 0.0151**
	VM‐SSIM ↑	0.8561 ± 0.0240	0.9660 ± 0.0071	0.8958 ± 0.0228	0.9565 ± 0.0163	0.9769 ± 0.0088	0.9816 ± 0.0064	**0.9872 ± 0.0056**
	NMSE ↓	0.2076 ± 0.0350	0.0922 ± 0.0146	0.1849 ± 0.0446	0.5467 ± 0.0918	0.0621 ± 0.0137	0.0543 ± 0.0107	**0.0434 ± 0.0082**

*Note*: The table shows PSNR, SSIM, VM‐SSIM, and NMSE, presented as mean ± SD across all subjects. The top‐performing method for each task and metric is highlighted in bold font.

### 3.2 Ablation studies

**FIGURE 5:**
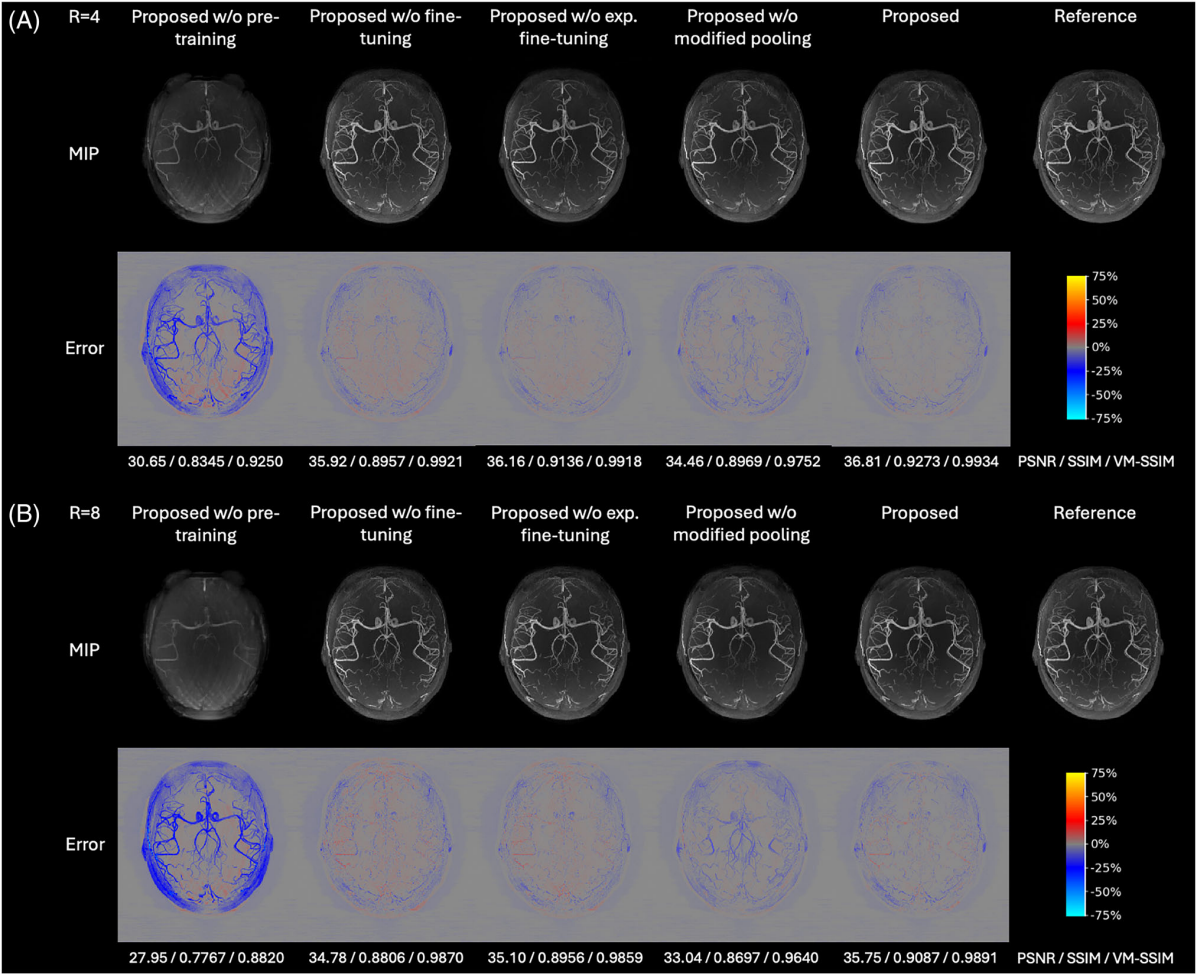
Axial MIP reconstructions of retrospectively undersampled experimental in vivo data for *R* = 4 (subfigure A) and *R* = 8 (subfigure B), showing results for methods: Proposed w/o pre‐training, Proposed w/o fine‐tuning, Proposed w/o exp. fine‐tuning, Proposed w/o modified pooling, and Proposed. The subfigure layout follows Figure 3.

To separately assess the contributions of pre‐training, fine‐tuning, data realism, and architectural modifications, representative MIP reconstructions and quantitative results for the ablation variants—Proposed w/o pre‐training, Proposed w/o fine‐tuning, Proposed w/o exp. fine‐tuning, and Proposed w/o modified pooling—and the final Proposed method are presented in Figure 5 and Table 2.

**TABLE 2:** Quantitative comparison of reconstruction performance on retrospectively undersampled experimental in vivo data for *R* = 4 and *R* = 8, showing results for methods in Figure 5.

Acceleration	Metric	Proposed w/o pre‐training	Proposed w/o fine‐tuning	Proposed w/o exp. fine‐tuning	Proposed w/o modified pooling	Proposed
*R* = 4	PSNR ↑	30.75 ± 1.37	35.39 ± 1.22	35.77 ± 1.42	34.40 ± 1.44	**36.39 ± 1.36**
	SSIM ↑	0.8288 ± 0.0238	0.8920 ± 0.0123	0.9058 ± 0.0117	0.8942 ± 0.0137	**0.9201 ± 0.0104**
	VM‐SSIM ↑	0.9151 ± 0.0147	0.9901 ± 0.0036	0.9906 ± 0.0042	0.9732 ± 0.0081	**0.9921 ± 0.0036**
	NMSE ↓	0.1217 ± 0.0216	0.0396 ± 0.0046	0.0381 ± 0.0052	0.0526 ± 0.0105	**0.0314 ± 0.0044**
*R* = 8	PSNR ↑	28.63 ± 1.59	34.13 ± 1.59	34.31 ± 1.61	32.21 ± 1.72	**35.12 ± 1.47**
	SSIM ↑	0.7828 ± 0.0290	0.8783 ± 0.0179	0.8875 ± 0.0162	0.8633 ± 0.0220	**0.9067 ± 0.0151**
	VM‐SSIM ↑	0.8773 ± 0.0213	0.9841 ± 0.0054	0.9852 ± 0.0062	0.9582 ± 0.0118	**0.9872 ± 0.0056**
	NMSE ↓	0.1999 ± 0.0437	0.0545 ± 0.0097	0.0537 ± 0.0102	0.0882 ± 0.0220	**0.0434 ± 0.0082**

*Note*: The table format follows Table 1.

Proposed w/o pre‐training exhibited significantly inferior reconstruction quality for both *R* = 4 and *R* = 8, highlighting the importance of the proposed pre‐training approach. Proposed w/o fine‐tuning, while achieving good performance, benefited further from fine‐tuning using just two single‐slab experimental datasets, resulting in improved reconstruction for both acceleration factors with reduced noise and higher PSNR, SSIM, and VM‐SSIM.

Proposed w/o exp. fine‐tuning, which used only the magnitude of the experimental data combined with simulated phase and coils, yielded better contrast and suppressed noise (appearing red in error maps) compared to the non‐fine‐tuned model. However, this improvement was not to the extent observed in the fully fine‐tuned Proposed model, which also reflected in the quantitative metrics: for *R* = 8, Proposed w/o exp. fine‐tuning increased PSNR by 0.18 dB, SSIM by 0.92%, and VM‐SSIM by 0.11%, compared to larger gains of 0.99 dB, 2.83%, and 0.31%, respectively, from the fully fine‐tuned model.

Proposed w/o modified pooling, which omitted the customized network downsampling and upsampling, showed a clear degradation in reconstruction fidelity. This was most apparent as loss of vascular structures and reduced vessel continuity, and was quantitatively supported by reductions in all image quality metrics.

### 3.3 Comparison of simulation methods

**FIGURE 6:**
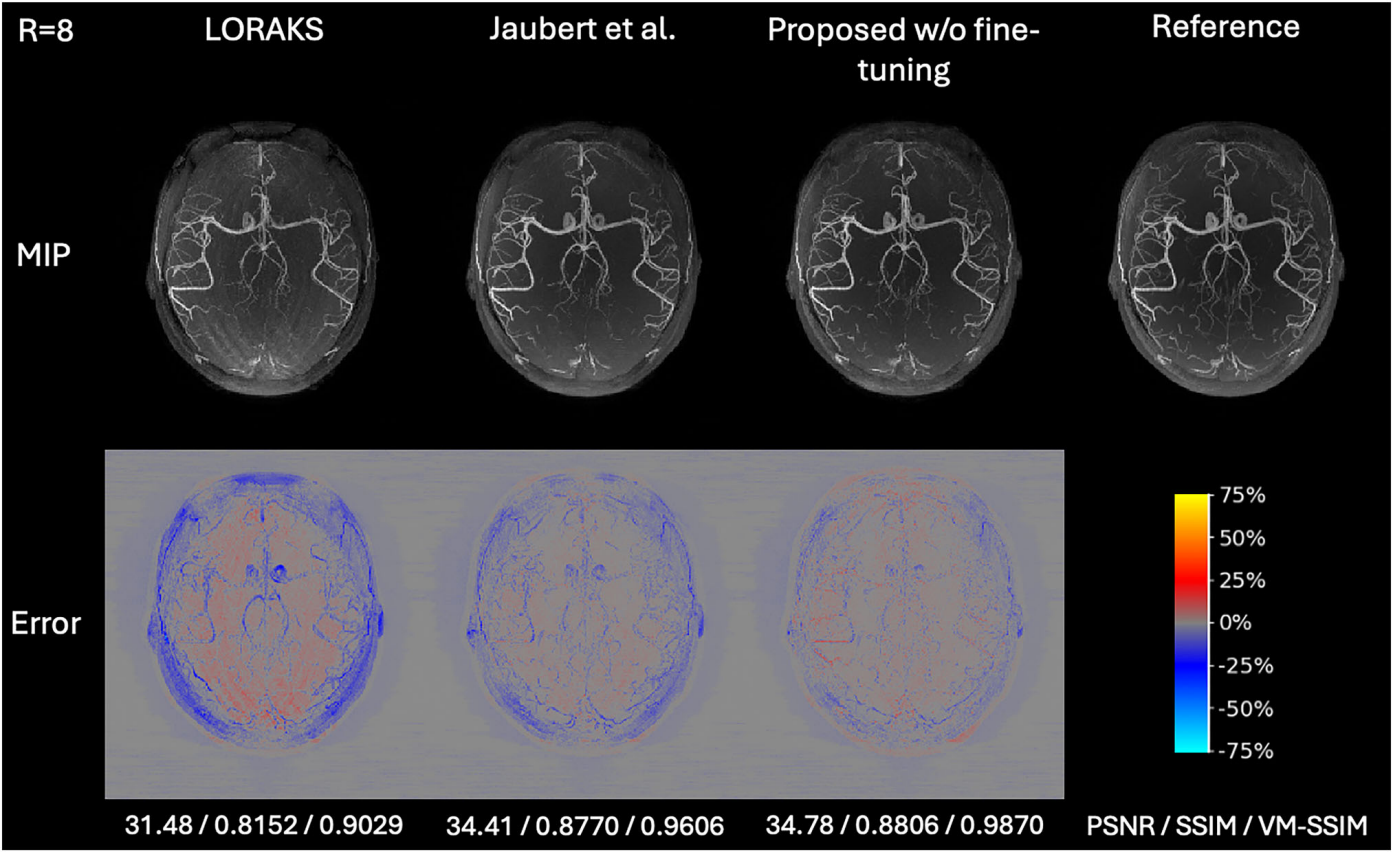
Axial MIP reconstructions of retrospectively undersampled experimental in vivo data for *R* = 8, showing results of the proposed 3D model trained on the dataset generated by the proposed data simulation pipeline and datasets where part of the pipeline was replaced with methods from LORAKS and Jaubert et al. The figure layout follows Figure 3.

To evaluate the impact of the proposed raw k‐space data simulation methods, MIP reconstructions and quantitative results of the proposed 3D model, trained on two alternative datasets that replaced part of the simulation pipeline with two adapted recent methods (LORAKS^42^ and Jaubert et al.^44^), are compared in Figure 6 and Table S3 (Supporting Information) for *R* = 8. The proposed data simulation pipeline demonstrated higher reconstruction quality compared to existing simulation methods, achieving the highest PSNR, SSIM, and VM‐SSIM.

### 3.4 Reconstructions of prospective datasets

**FIGURE 7:**
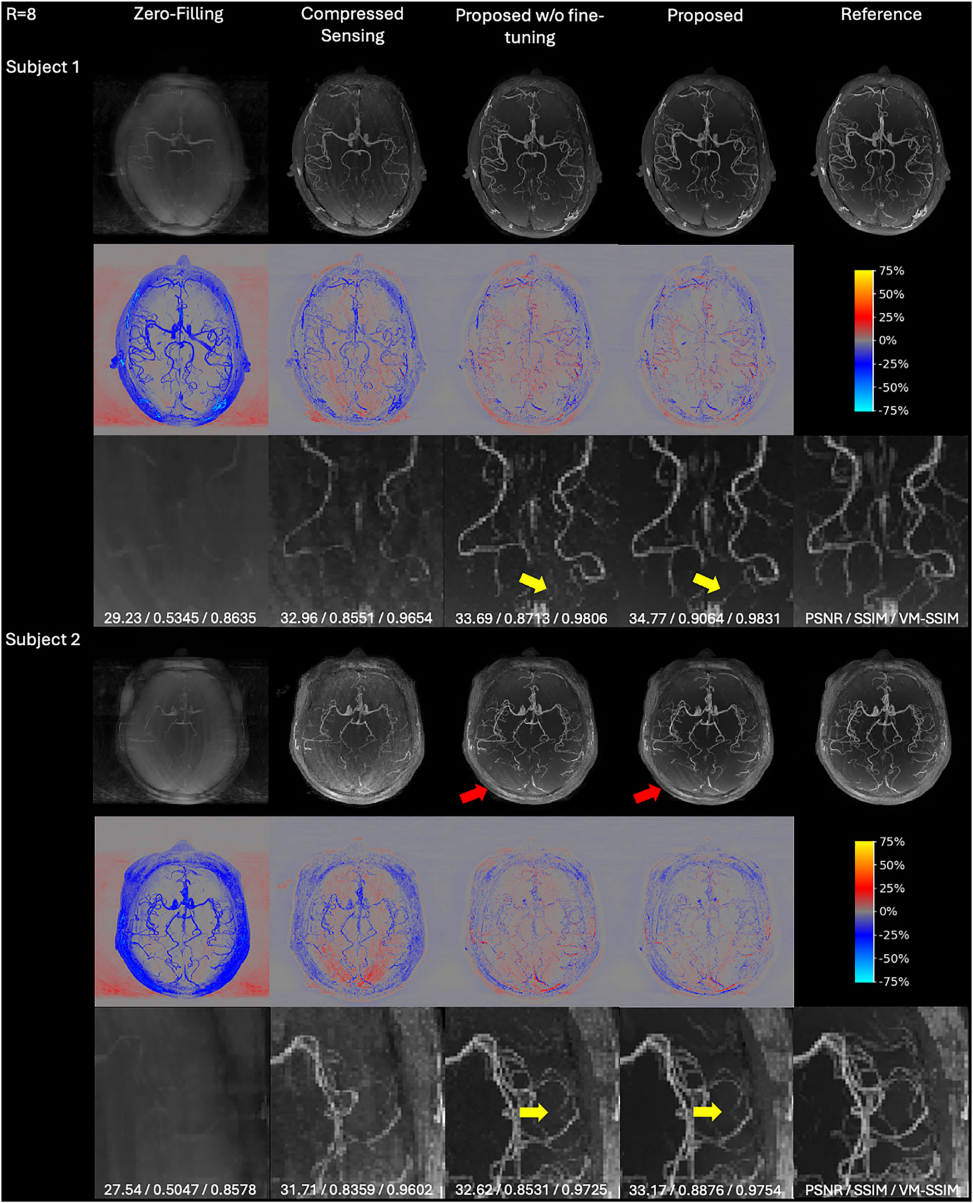
Axial MIP reconstructions of prospectively undersampled experimental in vivo data for *R* = 8 with zoomed views, showing results for Zero‐Filling, L1 wavelet‐regularized CS, Proposed w/o fine‐tuning, and Proposed methods. For each subject, the first row presents reconstructed MIP angiograms, the second row shows error maps, the third row provides zoomed views, and PSNR/SSIM/VM‐SSIM metrics are reported at the bottom. Yellow arrows indicate improvements due to single‐slab fine‐tuning, while red arrows highlight slight residual aliasing.

To evaluate the performance and robustness of the proposed methods in a real‐world scenario, reconstruction results of the Proposed and Proposed w/o fine‐tuning methods on prospectively eight‐fold accelerated datasets were compared against Zero‐Filling, L1 wavelet‐regularized CS, and fully sampled references, as shown in Figure 7 and Table 3. While registration was performed using FLIRT to mitigate inter‐scan misalignment between prospectively undersampled and fully sampled acquisitions, residual misregistration persists due to the difficulty of sub‐voxel transforms (rotation under 0.05° and translation below 0.2 mm), scanner drift, and possible physiological variations between scans. These slight misalignments are visible in the error maps and led to slightly lower quantitative metrics compared to the retrospective study.

**TABLE 3:** Quantitative comparison of reconstruction performance on prospectively undersampled experimental in vivo data for *R* = 8, showing results for methods in Figure 7.

Acceleration	Metric	Zero‐filling	CS	Proposed w/o fine‐tuning	Proposed
*R* = 8	PSNR ↑	28.54 ± 0.95	32.65 ± 0.85	32.96 ± 0.88	**33.81 ± 0.83**
	SSIM ↑	0.5067 ± 0.0409	0.8396 ± 0.0167	0.8642 ± 0.0150	**0.8939 ± 0.0114**
	VM‐SSIM ↑	0.8636 ± 0.0137	0.9617 ± 0.0038	0.9776 ± 0.0045	**0.9792 ± 0.0040**
	NMSE ↓	0.2188 ± 0.0306	0.0846 ± 0.0080	0.0776 ± 0.0068	**0.0647 ± 0.0055**

*Note*: The table format follows Table 1.

Compared to CS, both proposed methods reconstructed more vessels with fewer artifacts and aliasing, consistent with the previous retrospective studies. Notably, the Proposed method, fine‐tuned using only two single slabs of experimental data, achieved the best quantitative performance with the highest PSNR, SSIM, and VM‐SSIM. As highlighted by the yellow arrows in Figure 7, single‐slab fine‐tuning reduced noise and made the small vessel signals more clearly visible, as evidenced by diminished red additive errors in the error maps and corresponding increases in VM‐SSIM. However, some very small vessels were still lost, and slight residual aliasing persisted in the posterior region of Subject 2, as indicated by the red arrows in Figure 7. Notably, this artifact was found only in Subject 2 and was not observed in the other cases. This might have been caused by motion or physiological variation during the prospective acquisition, as similar degradation was also observed in CS reconstructions for Subject 2.

## 4 DISCUSSION

The proposed few‐shot learning approach for highly accelerated 3D TOF‐MRA, integrating a novel raw k‐space data simulation method with an adapted 3D variational network model, effectively reconstructed high‐quality 3D TOF‐MRA images from undersampled in vivo acquisitions relying on only two single‐slab experimentally acquired raw k‐space datasets for fine‐tuning and validation. It maintained high image fidelity even under more aggressive undersampling (8×), with rapid reconstruction times (15 s for all slabs). The two fine‐tuning datasets were each acquired in under 2 min, and the entire fine‐tuning process completes in approximately 10 min on a single GPU. Despite this minimal data and time requirement, the few‐shot fine‐tuning led to considerable improvements in reconstruction quality, making the approach practical for rapid deployment in real‐world settings, particularly compared to the cost and effort of collecting extensive local datasets and training from scratch.

In retrospective studies, the proposed method outperformed all other tested reconstruction methods in reconstructing retrospectively undersampled experimental in vivo 3D TOF‐MRA data, particularly at higher acceleration factors, which exhibited the least noise, artifacts, and loss of details, with large differences from the ground truth only in non‐critical peripheral regions such as the ears and skull. In contrast, the 2D E2E‐VarNet pre‐trained solely on the fastMRI dataset with T1w, T2w, and FLAIR images exhibited persistent aliasing even after fine‐tuning. However, the 2D E2E‐VarNet pre‐trained on simulated 3D TOF‐MRA raw k‐space datasets achieved much less aliasing and higher image quality, which underscored the value of the proposed few‐shot learning framework for robust generalization. However, this advantage did not extend to DPI‐Net, which may be attributed to its direct image‐to‐image mappings without incorporating MRI physics models, making it more susceptible to overfitting.

The effectiveness of the proposed 3D model, incorporating architectural modifications tailored to 3D TOF‐MRA, was evident when compared to the 2D E2E‐VarNet. The customized 3D model leveraged both inter‐ and intra‐slice correlations for continuous vessel depiction and was specifically designed to better handle 3D thin‐slab inputs with partial Fourier, underscoring the importance of modality‐specific model adaptations.

Ablation studies demonstrated that the proposed pre‐trained model already achieved strong performance, and fine‐tuning using just two single‐slab experimental in vivo datasets further improved reconstruction quality by effectively reducing aliasing and noise. This highlights the potential generalizability and practical utility of the proposed approach to new scanners, protocols, or hardware upgrades—any changes that alter data distribution—where the pre‐trained model can be efficiently adapted by re‐tuning using only two single‐slab datasets, thereby avoiding the need to acquire large local datasets each time such changes occur. While further fine‐tuning with larger datasets might yield additional performance gains, it would come at the cost of increased acquisition and computational burden, which this work aims to minimize. Additionally, the studies underscored the value of the proposed few‐shot learning framework in data‐limited scenarios, as training an effective reconstruction model from scratch using only very limited experimentally acquired data proved infeasible.

Notably, fine‐tuning using only the magnitude component of the experimental data while relying on simulated phase and coil sensitivity maps provided partial improvement over the non‐fine‐tuned baseline, particularly in terms of reduced noise and improved contrast. This suggests that, while site/scanner‐specific magnitude images can help reduce domain mismatch and enhance reconstruction robustness, the absence of realistic, scanner‐specific phase and coil sensitivity information still significantly constrains performance. The results indicate that incorporating complete experimental data is important for realizing the full benefits of fine‐tuning, especially in phase‐sensitive applications like TOF‐MRA. Furthermore, ablating the customized down‐/up‐sampling design in the model architecture resulted in visibly lost vascular detail and reduced vessel continuity. This highlights again the importance of modality‐specific architectural adaptations in preserving vascular information.

Compared to other raw k‐space data simulation techniques, the proposed simulation method enabled the model to reconstruct 3D TOF‐MRA images with higher quality and fewer artifacts. This demonstrates the ability of the proposed data simulation pipeline to generate more realistic raw k‐space data for 3D TOF‐MRA through its more complex and curated simulation processes, which account for high‐frequency variations inherent to its flow‐related enhancement nature. The proposed pipeline demonstrated a more significant improvement over existing methods in phase simulation, which is inherently more challenging than coil sensitivity simulation due to the absence of smooth and consistent profiles across MRI modalities. Therefore, enhancing phase simulation is likely to be more critical, and the new method introduced here, with broad parameter ranges, effectively captures the necessary diversity in phase profiles.

When tested on prospectively undersampled datasets, the proposed method consistently demonstrated high‐quality reconstructions. Compared to the benchmark method, CS, the proposed method reconstructed more fine vessels with markedly less noise and aliasing. The robust and consistent performance of the proposed method on retrospective studies and prospectively undersampled datasets—similar to real‐world scenarios—can be attributed to the use of large amounts of multi‐center, multi‐vendor training magnitude images and the proposed randomized data simulation with wide parameter ranges. Additionally, the benefit of fine‐tuning the proposed method using only two single‐slab experimental in vivo datasets was also seen in prospectively undersampled data.

Despite these strengths, some limitations remain. First, for the acceleration factor of 8, although the proposed method significantly outperformed other methods, some very small vessel signals were missed in the reconstructions, particularly for the method without fine‐tuning. This may be due to a residual domain shift between the simulated and experimental acquired data, which could potentially be addressed by a more rigorous optimization of all parameters within the data simulation pipeline or by incorporating additional data sources for better generalization. Second, when data quality is low—such as in cases of poor SNR or significant motion corruption—some aliasing remains in the reconstructions. This issue could be mitigated by incorporating simulations of motion corruption and employing other data augmentation techniques in future work. Third, the current method employs fixed‐size convolutional kernels, which may limit performance when test data differ substantially in spatial resolution from the training data. While the proposed method demonstrated robustness to moderate variations in imaging parameters, improving generalizability across a broader range of resolutions remains an important direction for future work. Fourth, the proposed method was evaluated on a limited healthy cohort, and further validation incorporating radiologic assessment is needed in a larger patient population with diverse cerebrovascular disorders to better assess its clinical utility. Finally, developing an inline reconstruction pipeline would be essential in future work to enhance the clinical applicability of the proposed methods.

In conclusion, this work has demonstrated the effectiveness of a new few‐shot learning‐based reconstruction method designed for highly accelerated 3D TOF‐MRA, which requires only minimal experimentally acquired data to achieve superior results on both retrospective and prospective in vivo data over existing methods with rapid reconstruction times. Given the challenges of collecting a large set of raw k‐space data for 3D TOF‐MRA, this method holds significant promise for advancing research and clinical applications of high‐resolution, whole‐head 3D TOF‐MRA imaging.

## FUNDING INFORMATION

Wellcome Trust, Grant/Award Numbers: 203139/Z/16/Z, 203139/A/16/Z, 220204/Z/20/Z; NIHR Oxford Biomedical Research Centre, Grant/Award Number: NIHR203311; NIHR Oxford Health Biomedical Research Centre, Grant/Award Number NIHR203316; Siemens Healthineers; Medical Research Council; Canada Research Chairs; Vivensa Foundation.

## CONFLICT OF INTEREST STATEMENT

Hao Li receives studentship support from Siemens Healthineers. Iulius Dragonu is an employee of Siemens Healthineers. Peter Jezzard is the Editor‐in‐Chief of Magnetic Resonance in Medicine. In line with COPE guidelines, Peter Jezzard recused himself from all involvement in the review process of this paper, which was handled by an associate editor. He and the other authors had no access to the identities of the reviewers.

## Supporting information


**Table S1.** Parameters for phase simulation. Values for $\sigma_{n_{j}}$ represent ratios relative to the maximum intensity, while values for $\sigma_{G_{j}}$ represent ratios relative to the size of the dimensions. 𝒰(a,b) denotes a uniform distribution within a range [a,b] from which the parameters are randomly sampled.
**Table S2.** Parameters for coil sensitivity map simulation. Values for $\sigma_{n_{j}}$ represent ratios relative to the maximum intensity, while values for $\sigma_{G_{j}}$, $\sigma_{x_{i}}$, $\sigma_{y_{i}}$, and $\sigma_{z_{i}}$ represent ratios relative to the size of the dimensions. 𝒰(a,b) denotes a uniform distribution within a range [a,b] from which the parameters are randomly sampled, while 𝒩(μ,σ) is a Gaussian distribution with mean μ and standard deviation σ.
**Table S3.** Quantitative comparison of reconstruction performance on retrospectively undersampled experimental in vivo data for *R* = 8, showing results for methods in Figure 6. The table format follows Table 1.
**Figure S1.** Training and validation loss during fine‐tuning of the proposed method.
**Figure S2.** Axial MIP reconstructions of retrospectively undersampled experimental in vivo data from another subject for acceleration factor *R* = 4, showing results for comparison methods alongside the fully sampled reference. The top row shows reconstructed MIP angiograms (greyscale‐adjusted for comparison), the second row shows error maps (percentage difference from the reference normalized by the maximum intensity), and PSNR/SSIM/VM‐SSIM metrics are reported at the bottom.
**Figure S3.** Axial MIP reconstructions of retrospectively undersampled experimental in vivo data from another subject for *R* = 8 with zoomed views. The first two rows follow Figure S2, the third and fourth rows are zoomed views of the first two rows, and PSNR/SSIM/VM‐SSIM metrics are shown at the bottom.

## Data Availability

The original IXI dataset used for training can be found here: https://brain‐development.org/ixi‐dataset/. The code underlying the phase and coil simulations and model training can be found here: https://github.com/HLi2000/FewShotTOF. Pre‐trained models and data underlying the tables in this study can be found here: https://doi.org/10.5281/zenodo.17077715. We are currently unable to share the full in vivo data due to data protection issues, although the Centre for Integrative Neuroimaging is actively working on a solution to this.
