# Supplementary material for: Few‐shot learning for highly accelerated 3D time‐of‐flight MRA reconstruction
Source: Magn Reson Med. 2025 Sep 10;95(2):770–86. doi: 10.1002/mrm.70072 (PMC12681298; doi:10.1002/mrm.70072)
Supplement: Supplementary file 1 — Table S1. Parameters for phase simulation. Values for σnj represent ratios relative to the maximum intensity, while values for σGj represent ratios relative to the size of the dimensions. 𝒰(a,b) denotes a uniform distribution within a range [a,b] from which the parameters are randomly sampled. Table S2. Parameters for coil sensitivity map simulation. Values for σnj represent ratios relative to the maximum intensity, while values for σGj, σxi, σyi, and σzi represent ratios relative to the size of the dimensions. 𝒰(a,b) denotes a uniform distribution within a range [a,b] from which the parameters are randomly sampled, while 𝒩(μ,σ) is a Gaussian distribution with mean μ and standard deviation σ. Table S3. Quantitative comparison of reconstruction performance on retrospectively undersampled experimental in vivo data for R = 8, showing results for methods in Figure 6. The table format follows Table 1. Figure S1. Training and validation loss during fine‐tuning of the proposed method. Figure S2. Axial MIP reconstructions of retrospectively undersampled experimental in vivo data from another subject for acceleration factor R = 4, showing results for comparison methods alongside the fully sampled reference. The top row shows reconstructed MIP angiograms (greyscale‐adjusted for comparison), the second row shows error maps (percentage difference from the reference normalized by the maximum intensity), and PSNR/SSIM/VM‐SSIM metrics are reported at the bottom. Figure S3. Axial MIP reconstructions of retrospectively undersampled experimental in vivo data from another subject for R = 8 with zoomed views. The first two rows follow Figure S2, the third and fourth rows are zoomed views of the first two rows, and PSNR/SSIM/VM‐SSIM metrics are shown at the bottom. [file MRM-95-770-s001.docx]

**Supporting Information for “Few-shot learning for highly accelerated 3D time-of-flight MRA reconstruction”**

Table S1. Parameters for phase simulation. Values for $\sigma_{n_{j}}$​​ represent ratios relative to the maximum intensity, while values for $\sigma_{G_{j}}$ represent ratios relative to the size of the dimensions. $\mathcal{U}\left( a,b \right)$ denotes a uniform distribution within a range $[a,b]$ from which the parameters are randomly sampled.

| $j$ | $\sigma_{n_{j}}$​​ | $\sigma_{G_{j}}$ |
| --- | --- | --- |
| 1 | $\mathcal{U}$(1.0, 3.0) | $\mathcal{U}$(0.005, 0.025) |
| 2 | $\mathcal{U}$(0, 0.01) | $\mathcal{U}$(0.025, 0.25) |
| 3 | $\mathcal{U}$(0, 0.0001) | 1.0 |

Table S2. Parameters for coil sensitivity map simulation. Values for $\sigma_{n_{j}}$​​ represent ratios relative to the maximum intensity, while values for $\sigma_{G_{j}}$, $\sigma_{x_{i}}$, $\sigma_{y_{i}}$, and $\sigma_{z_{i}}$ represent ratios relative to the size of the dimensions. $\mathcal{U}\left( a,b \right)$ denotes a uniform distribution within a range $[a,b]$ from which the parameters are randomly sampled, while $\mathcal{N(}\mu,\sigma)$ is a Gaussian distribution with mean $\mu$ and standard deviation $\sigma$.

| $j$ | $\sigma_{n_{j}}$​​ | $\sigma_{G_{j}}$ | $\sigma_{x_{i}}$​​ | $\sigma_{y_{i}}$​​ | $\sigma_{z_{i}}$​​ |
| --- | --- | --- | --- | --- | --- |
| 1 | $\mathcal{U}$(0, 0.25) | 0.0075 | $\mathcal{N}$(0.25, 0.05) | $\mathcal{N}$(0.25, 0.05) | $\mathcal{N}$(0.25, 0.05) |
| 2 | $\mathcal{U}$(0, 0.0001) | $1.0$ |  |  |  |


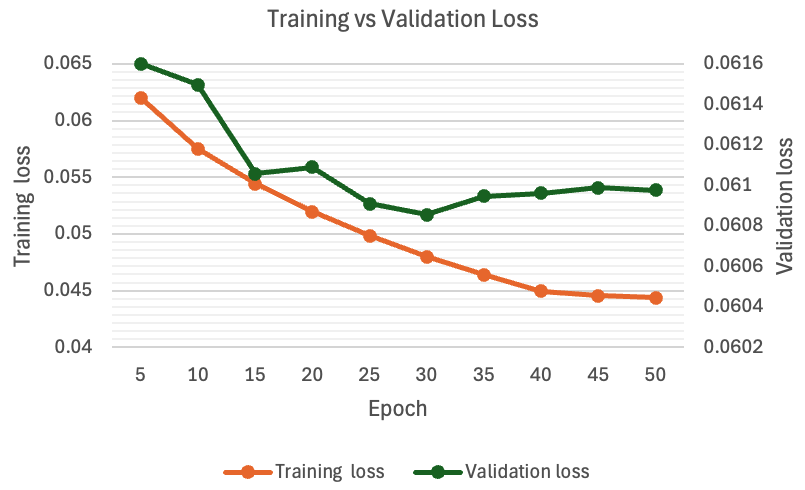


Figure S1. Training and validation loss during fine-tuning of the proposed method.


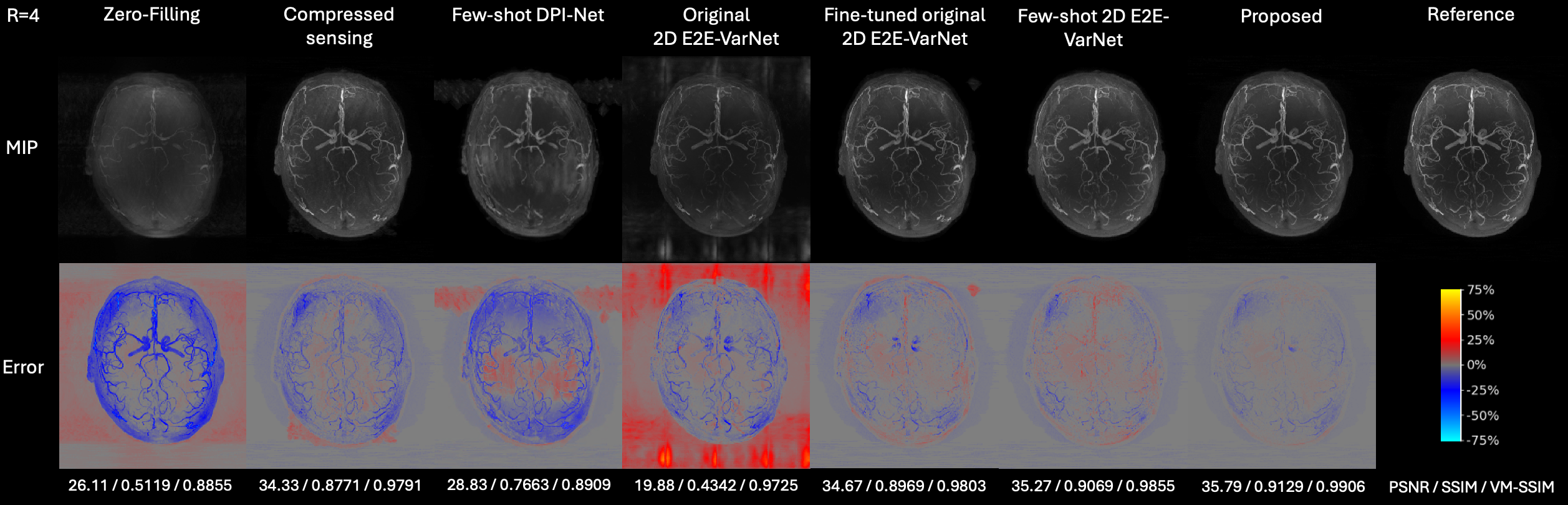


Figure S2. Axial MIP reconstructions of retrospectively undersampled experimental in vivo data from another subject for acceleration factor R=4, showing results for comparison methods alongside the fully sampled reference. The top row shows reconstructed MIP angiograms (greyscale-adjusted for comparison), the second row shows error maps (percentage difference from the reference normalized by the maximum intensity), and PSNR/SSIM/VM-SSIM metrics are reported at the bottom.


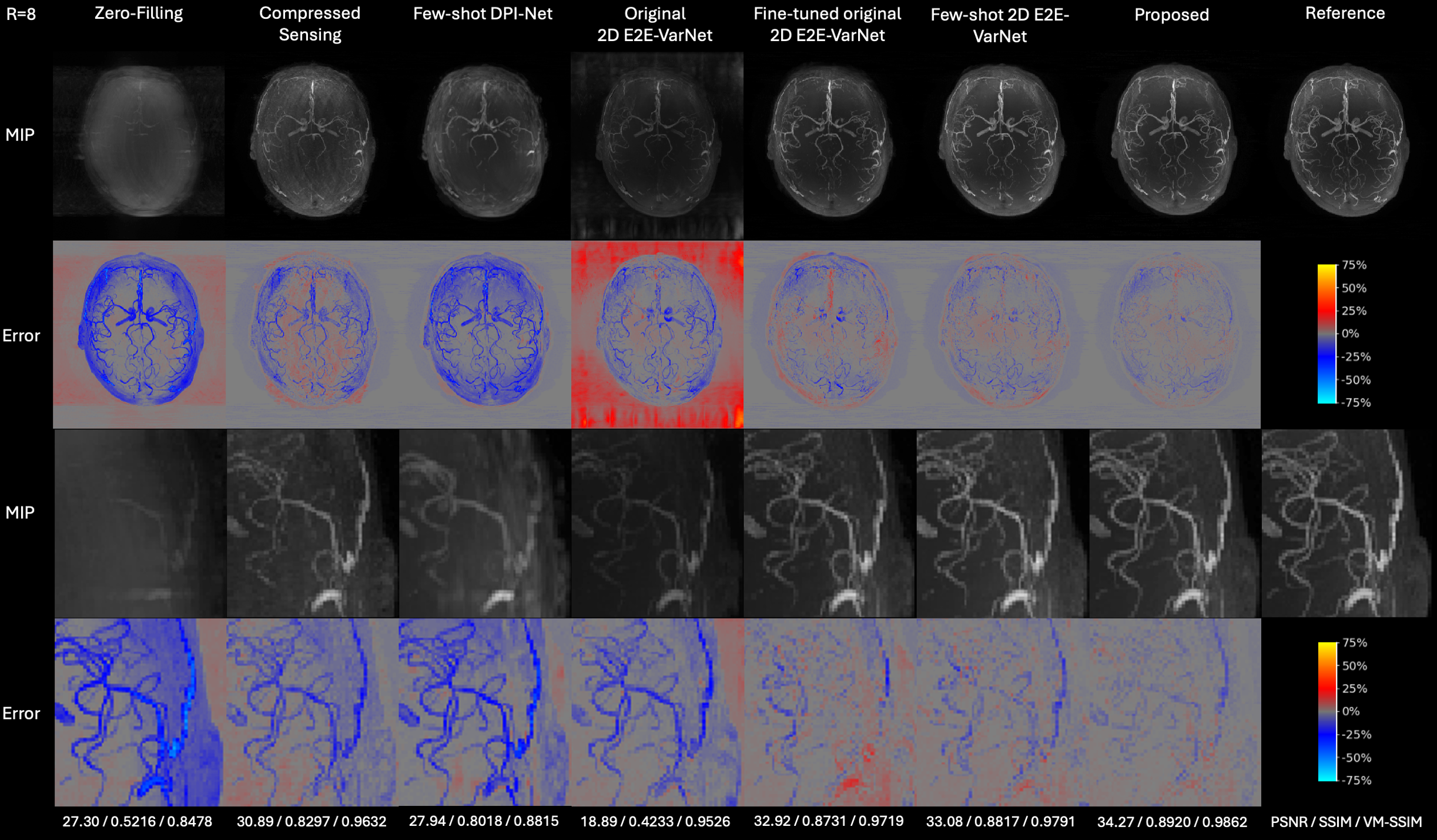


Figure S3. Axial MIP reconstructions of retrospectively undersampled experimental in vivo data from another subject for R=8 with zoomed views. The first two rows follow Figure S2, the third and fourth rows are zoomed views of the first two rows, and PSNR/SSIM/VM-SSIM metrics are shown at the bottom.

Table S3. Quantitative comparison of reconstruction performance on retrospectively undersampled experimental *in vivo* data for R=8, showing results for methods in Figure 6. The table format follows Table 1.
